# Factors associated with mortality in intracranial infection patients admitted to pediatric intensive care unit: A retrospective cohort study

**DOI:** 10.1016/j.amsu.2021.102884

**Published:** 2021-09-23

**Authors:** Asmaul Kholifia, Desy Rusmawatiningtyas, Firdian Makrufardi, Ida Safitri Laksanawati, Intan Fatah Kumara, Nurnaningsih

**Affiliations:** Department of Child Health, Faculty of Medicine, Public Health and Nursing, Universitas Gadjah Mada/Dr. Sardjito Hospital, Yogyakarta, 55281, Indonesia

**Keywords:** Predictor factor, Intracranial infection, Mortality, Pediatric intensive care unit

## Abstract

**Background:**

Intracranial infection is a major cause of emergency and death in children. To assist clinical decision-making in patient management, we conducted a study about factors associated with mortality. This study aimed to evaluate factors associated with mortality in pediatric patients with intracranial infection.

**Methods:**

We performed a cohort retrospective study in our tertiary hospital to evaluate the outcomes of patients admitted to the pediatric intensive care unit (PICU) from 2014 to 2018. The Chi-square test was performed to determine the significance of the predictor, and *p* < 0.05 was considered to indicate a statistically significant result. We used multivariate logistic regression to determine relative risk (RR) with 95% confidence interval (CI).

**Results:**

We recruited 112 patients who were admitted to the PICU of our tertiary hospital. A total of 38.4% were diagnosed with encephalitis, 9.8% meningitis and 51.8% meningoencephalitis. Of the 112 patients who met the inclusion criteria, 28 (25%) patients died in the PICU. The need of mechanical ventilation support variable had a statistically significant association with mortality (RR 22**.**76; 95% CI: 3**.**88–51.45).

**Conclusion:**

Recognition of conditions that exacerbate intracranial infection in children needs to be done as early as possible. Moreover, the need of mechanical ventilation support in the PICU needs more attention.

## Highlights

• Intracranial infection is a major cause of emergency and death in children.
• Recognition of conditions that exacerbate intracranial infection.
• The need of mechanical ventilation support in the pediatric intensive care unit.

## 1 Introduction

Intracranial infection is a life-threatening infection that can affect the meninges or the brain. Infections of the central nervous system are caused by various microorganisms with clinical manifestations such as meningitis, encephalitis, and pyogenic infections such as empyema and brain abscess [1]. According to a study in 2009, the most common intracranial infection in infants and children is meningitis. The incidence of bacterial meningitis is high in the first few months of life and continues to be high until the age of two, after which it declines significantly [2].

Encephalitis is a medical emergency that requires prompt diagnosis and specific therapy. The incidence of acute encephalitis varies worldwide but is generally between 3.5 and 7.4 cases per 100,000 patients per year [3].

The encephalitis mortality rate in Indonesia is ranked 14th in the Asian region, namely 1.2 cases per 100,000 population, which was about 38.3% since 1990 with an average of 1.7% per year. The highest mortality rate occurred in pediatric patients aged 1–4 years, namely 4 cases per 100,000 in boys and 2.6 cases per 100,000 in girls [4].

Research on factors associated with mortality from intracranial infection in children is rarely done. Several studies have been done specifically related to intracranial infection, encephalitis, meningitis, brain abscess or other intracranial infections. Research on factors associated with mortality in encephalitis has been done before and one study stated that the factors influencing mortality in pediatric patients with acute encephalitis who were admitted to the pediatric intensive care unit (PICU) were hyponatremia and patients who were in shock and received inotropes on admission [5]. Research conducted in adult patient subjects found that death in encephalitis was associated with cerebral edema, epileptic state and thrombocytopenia [6]. Meanwhile, another research in pediatric patients with acute encephalopathy stated that factors associated with mortality were recurrent seizures, Glasgow Coma Scale (GCS) < 8, shock, severe anemia, and bradycardia [7]. Therefore, we aimed to evaluate factors associated with mortality in pediatrics with intracranial infection admitted to PICU in our low-resource setting.

## 2 Methods

### 2.1 Patients and study design

We performed a cohort retrospective study in our tertiary hospital to evaluate outcomes of pediatric patients admitted to the PICU. We collected patients' data from January 2014 to June 2018 who had been diagnosed with intracranial infection. All patients were followed-up until they received PICU outcome as either "survive" or "death". Included patients were children aged 1 month - 18 years who were admitted to PICU with intracranial infection including encephalitis, meningitis, and meningoencephalitis with a minimum length of stay of 1 day. We excluded patients with incomplete data in their medical records. This study was approved by the Medical and Health Research Ethics Committee of the Faculty of Medicine, Public Health and Nursing, Universitas Gadjah Mada, Yogyakarta, Indonesia: KE/FK/0707/EC/2018. The study was registered at the “Research Repository Faculty of Medicine, Public Health and Nursing, Universitas Gadjah Mada” with unique identifiying number UIN of 202108101. This study has been reported in line with PROCESS criteria [21].

### 2.2 Data collection

We recorded age, gender, nutritional status, head size, diagnosis and PICU length of stay at PICU. Epidemiological and clinical characteristics were among the potential factors. We evaluated potential factors that might be associated with mortality including: GCS, the need of mechanical ventilation support, status epilepticus, cerebral edema, anemia, and hyponatremia.

We defined age as the patient's age when being diagnosed with intracranial infection at the PICU. Gender was divided into male and female. Nutritional status was classified using the World Health Organization (WHO) growth chart: Weight-for-Height curve for children younger than 5 years old or body mass index (BMI)-for-age curve for children 5 years old or older. Data were plotted according to the WHO growth chart and categorized as obese, overweight, normoweight, underweight and malnutrition [8].

We categorized GCS to <8 and ≥ 8. We defined anemia based on laboratory results and the 2011 WHO recommendation for anemia diagnosis. Children were categorized as having anemia and non-anemia based on their age [9]. Cerebral edema was a condition of cerebral edema in patients that can be seen from the computerized tomography (CT) scan image and stated in the medical records [10]. Hyponatremia was a condition where the sodium concentration was beyond the normal limit in the results of blood electrolyte examination, which was <135 mmol/L on the first electrolyte level examination performed in the PICU [11].

### 2.3 Outcome measures

The follow-up period finished when each patient's PICU result was determined, which was either survive or death. The mortality rate of intracranial infection was defined as the proportion of patients admitted to the PICU and died during hospitalization in the study period.

### 2.4 Data analysis

Independent variables in each subject were analyzed using SPSS Statistics 23rd version (IBM Corp., Armonk, NY). The Chi-square test was performed to determine the significance of the predictor, and *p* < 0.05 was considered to indicate a statistically significant result. We used multivariate logistic regression to determine factors associated with mortality.

## 3 Results

We identified 112 patients who were admitted to the PICU with a diagnosis of encephalitis, meningitis, and meningoencephalitis. The basic data characteristics of patients with intracranial infections analyzed in this study are shown in Table 1. A total of 38.4% were diagnosed with encephalitis, 9.8% meningitis and 51.8% meningoencephalitis. Of the 112 patients who met the inclusion criteria, 28 (25%) patients died in the PICU. The median length of stay in the PICU was 17 days with a minimum of 1 day and a maximum of 85 days.

**Table 1:** Clinical characteristics of pediatric patients with intracranial infection.

		Outcome		Total (n = 112)
		Death	Survive	
Age, n (%)	<1 year	13 (37.1%)	22 (62.9%)	35 (31.3%)
	1–5 year	11 (25.6%)	32 (74.4%)	43 (38.4%)
	>5 year	4 (11.7%)	30 (88.3%)	34 (30.3%)
Gender, n (%)	Male	14 (25.4%)	41 (74.6%)	55 (49.1%)
	Female	14 (24.6%)	43 (75.4%)	57 (50.9%)
Nutritional status, n (%)	Obese	2 (66.7%)	1 (33.3%)	3 (2.7%)
	Overweight	0 (0.0%)	4 (100%)	4 (3.6%)
	Normoweight	16 (22.2%)	55 (77.8%)	71 (63.4%)
	Underweight	3 (17.6%)	14 (82.4%)	17 (15.2%)
	Malnutrition	7 (41.2%)	10 (58.8%)	17 (15.2%)
Head size, n (%)	Microcephaly	10 (38.4%)	16 (61.6%)	26 (23.2%)
	Normocephaly	16 (19.3%)	67 (80.7%)	83 (74.1%)
	Macrocephaly	2 (66.7%)	1 (33.3%)	3 (2.7%)
Diagnosis, n (%)	Encephalitis	14 (32.5%)	29 (67.5%)	43 (38.4%)
	Meningitis	0 (0.0%)	11 (100%)	11 (9.8%)
	Meningoencephalitis	14 (24.1%)	44 (75.9%)	58 (51.8%)
PICU duration (days), median (min-max)		7 (1–42)	19 (1–85)	17 (1–85)

Min-max: minimum-maximum; PICU, pediatric intensive care unit.

Blood culture produced the most growth of microorganisms in 18 patients and 60% of them died, followed by cerebrospinal fluid (CSF) in 10 patients with 70% of those patients who died. The four bacterial pathogens that we found the most in our study were *Klebsiella pneumoniae, Staphylococcus haemolyticus, Pseudomonas aeruginosa* and *Streptococcus viridans* (Table 2).

**Table 2:** Outcomes of patients based on type of bacterial culture sample and microorganism.

Bacterial culture sample	Death (n)	Survive (n)	Total (n)
Blood	12	6	18
Cerebrospinal fluid	7	3	10
Endotracheal tube	1	3	4
Sputum	7	2	9
Urine	0	2	2
Catheter tip	1	0	1
Ear swab	1	0	1
Pharyngeal swab	1	0	1
Feces	0	2	2
Tracheal aspirate	0	2	2
Microorganism	Death (n)	Survive (n)	Total (n)
*Staphylococcus aureus*	3	0	3
*Acinetobacter baumanii*	1	2	3
*Staphylococcus haemolyticus*	2	4	6
*Pseudomonas aeruginosa*	4	2	6
*Enterobacter cloacae*	2	0	2
*Coagulase-negative staphylococci*	2	3	5
*Streptococcus viridans*	5	1	6
*Burkholderia cepacia*	4	1	5
*Klebsiella pneumoniae*	5	4	9
*Micrococcus luteus*	1	0	1
*Escherichia coli*	1	2	3
*Kocuria rosea*	1	0	1
*Kocuria kristinae*	0	1	1
*Cronobacter sakazakii*	1	1	2
*Staphylococcus epidermidis*	1	0	1
*Staphylococcus hominis*	0	1	1
*Staphylococcus warneri*	1	0	1

Bivariate analysis using Chi-square test showed that 3 variables had p value < 0.25 that were GCS variable with *p*-value of 0.064, anemia with *p*-value of 0.195 and the need of mechanical ventilation support with *p*-value of 0.004 (Table 3). Next, we analyzed those three variables’ association with mortality using logistic regression. We found that the need of mechanical ventilation support variable had a statistically significant association with mortality (RR 22**.**76; 95% CI: 3**.**88–51.45) (Table 4).

**Table 3:** Bivariate analyses of factors associated with mortality.

Variables		Outcome		*p*	RR	95% CI
		Death (n)	Survive (n)			
GCS	<8	18	37	0.064	1.86	0.95–3.67
	>8	10	47			
The need of mechanical ventilation support	Yes	28	64	0.004	0.69	0.60–0.79
	No	0	20			
Status epilepticus	Yes	10	33	0.736	0.89	0.45–1.74
	No	18	51			
Cerebral edema	Yes	9	34	0.432	0.76	0.37–1.52
	No	19	50			
Anemia	Yes	22	55	0.195	1.6	0.74–3.74
	No	6	29			
Hyponatremia	Yes	15	42	0.743	1.11	0.58–2.11
	No	13	42			

GCS: Glasgow Coma Scale, RR: Relative risk, CI: Confidence interval.

**Table 4:** Multivariate analyses of factors associated with mortality.

Variables	*P*	RR	95% CI
GCS	0.096	2.07	0.87–4.89
The need of mechanical ventilation support	0.003	22.76	3.88–51.45
Anemia	0.382	1.52	0.59–3.89

GCS: Glasgow Coma Scale, RR: Relative risk, CI: Confidence interval.

## 4 Discussion

In this study, the mortality rate of patients with intracranial infections in children at our tertiary referral hospital was 25%. This mortality rate is smaller than the mortality rate from acute encephalopathy reported in India (36.67%) [5] and the mortality rate from bacterial meningitis reported in Mexico in 2003 (33%) [12]. The mortality rate in our study was not much different from a previous study in the same setting, Dr. Sardjito General Hospital in 2014 which amounted to 22.1% [13].

The incidence of mortality in intracranial infection cases in the age group <5 years was 85%, which was high compared to the age group >5 years. This finding is in accordance with the 2013 study, where the incidence of patients who died at the age of <5 years, which was 65%, had a poor outcome and a high risk of mortality. The previous study stated that 50% of the types of diseases suffered by the age group <5 years were included in the types of diseases that could be prevented by giving vaccines [14].

The incidence of intracranial infection mortality according to gender in this study was not different. This finding is not in accordance with the data in Yogyakarta, Indonesia from a study conducted in 1999 where the incidence of intracranial infections in males was more than in females with a ratio of 3:1 [15]. Another study also found that the male patients had a higher risk for having meningitis [16].

In this study, blood cultures showed the most growth of microorganisms in 18 patients and 60% of them died. For CSF examination, only 10 patients showed the growth of microorganisms. Types of microorganisms that grew in CSF culture were *Streptococcus* spp.*, Micrococcus luteus, Coagulase-negative staphylococci, Escherichia coli, Staphylococcus epidermidis, Staphylococcus aureus, Staphylococcus hominis and Staphylococcus haemolyticus*. In a 2012 study, the microorganisms that were found to be causes of intracranial infections were *Neisseria meningitidis* (32.6%) and *Streptococcus pneumoniae* (27.9%) [6]. Another study stated that neurosurgery patients who underwent head surgery such as VP shunt placement were at risk for bacterial meningitis caused by bacteria from *Staphylococcus* strains, especially coagulase negative and gram negative bacteria [16].

In this study, GCS <8 was not a significant factor of mortality. This finding is different from the results of previous studies that indicated GCS <8 as a significant prognostic factor for mortality in patients with acute encephalopathy with OR 4.32 (95% CI: 1.07–7.40) [7]. GCS on admission to the hospital is an important predictor of intracranial infection, especially bacterial meningitis. Once the GCS drops to <13, the chances of saving the child and preventing neurological sequalae diminish very rapidly [17].

Anemia in our study was not a significant factor of death. Previous study found anemia as a predictor of death in encephalopathy with OR 4.3 (95% CI: 1.60–11.70), p = 0.004 [7]. Hemoglobin plays an important role in the process of transporting O_2_ throughout the body, and every 1 g of hemoglobin will bind 1.39 ml of O_2_ per dL of blood, thus the lower the hemoglobin, the less O_2_ will be transported throughout the body [18]. Pediatric patients admitted to the PICU are at great risk for anemia due to the underlying disease and its severity, iatrogenic blood loss, hemodilution, inadequate nutrition and slow bone marrow response. The results of one study showed an increase in the mortality rate of patients with hemoglobin levels less than 5 g/dL [19]. Anemia was not a significant factor in our study because the cut-off value for anemia was still high (11–13 g/dL) according to the 2011 WHO criteria, thus the associated mortality outcome was low.

In our study, 82% of patients were mechanically ventilated with a 100% mortality rate. This figure is higher than the study in 2013 which was 46% with a 95% mortality rate in adult patients treated with intracranial infections in the ICU [6]. Patients who had been on mechanical breathing for more than 48 h had a higher mortality rate as the number of comorbidities increased. Patients with a lower prehospitalization functional status also had a greater mortality rate [20].

Nevertheless, our study focused on mortality in pediatric patients with intracranial infection since there was no publication in Indonesia that discussed it with predictor factors [19]. Our study can also be a reference for better and comprehensive patient management; thus, more improved PICU management can reduce patient mortality. Moreover, we recommend physician who treated such patients should notice these factors. However, this study analyzes intracranial infection patients in a low-resource setting which might not reflect patients in a high-resource setting. A further retrospective multicenter study that includes other confounders is essential to provide more realistic survival data on PICU patients with intracranial infections.

## 5 Conclusions

Recognition of conditions that exacerbate intracranial infection in children needs to be done as early as possible. Moreover, the need of mechanical ventilation support in the PICU needs more attention due to being a significant factor of mortality in pediatric patients with intracranial infections.

## Funding

This research did not receive any specific grant from funding agencies in the public, commercial, or not-for-profit sectors.

## Consent

Written informed consent was obtained from the parents before joining the study. A copy of the written consent is available for review by the Editor-in-Chief of this journal on request.

## Provenance and peer review

Not commissioned, externally peer reviewed.

## Declaration of competing interest

No potential conflict of interest relevant to this article was reported.

## Registration of research studies

Research Repository Faculty of Medicine, Public Health and Nursing, Universitas Gadjah Mada Register Unique Identifying Number (UIN): 202108101.
